# Molecular chaperone effects on recombinant yield and binding characteristics of an ABA-specific scFv in *Escherichia coli*

**DOI:** 10.3389/fbioe.2025.1643833

**Published:** 2025-10-09

**Authors:** Shimiao Chen, Bin Shan, Yican Luo, Ganhui Mo, Usman Rasheed, Lilan Lv, Xinyu Yang, Qinyu Lu

**Affiliations:** ^1^ Key Laboratory, Guangxi Subtropical Crops Research Institute, Nanning, China; ^2^ Key Laboratory of Quality and Safety Control for Subtropical Fruit and Vegetable, Ministry of Agriculture and Rural Affairs, Nanning, China; ^3^ Guangxi Key Laboratory of Quality and Safety Control for Subtropical Fruits, Guangxi Subtropical Crops Research Institute, Nanning, China; ^4^ College of Food and Quality Engineering, Nanning University, Nanning, China

**Keywords:** ABA-scFv, molecular chaperone, *Escherichia coli*, soluble expression, antibody specificity

## Abstract

**Introduction:**

Recombinant single‐chain variable fragments (scFvs) are promising antibody formats for cost‐effective and scalable production. However, their soluble expression in Escherichia coli is often limited by misfolding and aggregation, particularly for scFvs targeting small molecule haptens such as abscisic acid (ABA). To address this bottleneck, molecular chaperones can be co‐expressed to enhance folding efficiency and functional yield.

**Methods:**

An ABA‐specific scFv was expressed in E. coli BL21(DE3) using five different chaperone plasmids (pG‐KJE8, pGro7, pKJE7, pG-Tf2, and pTf16). Soluble expression was quantified by His‐tag ELISA, and protein identity was confirmed by SDS‐PAGE and Western blot. Functional characterization included competitive ELISA for IC_50_ and specificity, while secondary structure was analyzed by FT‐IR and circular dichroism spectroscopy.

**Results:**

Trigger Factor (pTf16) significantly improved soluble scFv yield (19.65%) compared to the control (14.20%). The pKJE7 system achieved the highest sensitivity with the lowest IC_50_, whereas the pTf16 system provided superior specificity and a broader detection range by minimizing cross‐reactivity. Structural analysis revealed that pKJE7‐assisted scFv closely matched the predicted β‐sheet content, correlating with high sensitivity, while pTf16‐assisted scFv avoided non-native α-helices, supporting enhanced specificity. Circular dichroism further demonstrated that pKJE7‐ and pTf16‐assisted scFvs exhibited conformational rigidity consistent with a lock-and-key binding mechanism.

**Discussion:**

This study highlights that molecular chaperone choice influences both structural fidelity and functional performance of ABA‐scFv in E. coli. While pKJE7 favors high sensitivity, pTf16 yields highly specific and structurally stable antibodies. These findings establish a practical basis for developing low‐cost ABA immunoassays with tailored performance for agricultural biotechnology.

## 1 Introduction

Recombinant antibodies are widely used in diagnostics, research, and therapeutics because of their specificity and modular nature. Historically, the production of monoclonal antibodies relied heavily on the murine ascites method. However, this traditional technique has significant and widely recognized limitations. Beyond the critical ethical concerns surrounding animal welfare, which conflict with the guiding “3Rs” principles of modern research, the method suffers from profound practical drawbacks. A primary issue is the inherent batch-to-batch variation in antibody yield, affinity, and specificity, which arises from the variable physiological and immune responses of individual animals. This inconsistency complicates the standardization of assays and compromises experimental reproducibility.

Furthermore, the purification of antibodies from ascites fluid is a complex and costly endeavor, as the target antibody is heavily contaminated with host immunoglobulins, albumin, and other proteins, necessitating extensive multi-step chromatography. These collective challenges have led to a shift toward *in vitro* recombinant platforms (Peltomaa et al., 2022; Jayakrishnan et al., 2024). Among these, single-chain variable fragments (scFv) have emerged as a versatile and practical antibody format. These engineered fragments, which consist of the variable heavy (VH) and light (VL) domains connected by a flexible peptide linker, successfully preserve the complete antigen-binding site of a full-size antibody within a much smaller molecule (approximately 25–30 kDa). This minimalist yet fully functional format is ideally suited for cost-effective and highly scalable production in microbial fermentation systems (Marco, 2025; Mansourian et al., 2025), thereby directly addressing the ethical, consistency, and cost limitations of traditional methods (Kussia and Tessema, 2024).

The advantages of scFv technology are particularly relevant in fields that require robust and deployable molecular detection tools, such as modern agricultural biotechnology. The phytohormone abscisic acid (ABA) is a central signaling molecule in plant biology, regulating key aspects of development, including seed dormancy, as well as mediating adaptive responses to various environmental stressors like drought, soil salinity, and temperature extremes (Mo et al., 2024). The ability to rapidly and accurately quantify ABA concentrations is therefore valuable; it provides a direct biochemical indicator of a plant’s stress status. Such data can guide precision agriculture practices, for instance, by optimizing irrigation schedules to conserve water while preventing yield loss. It can also be used as a tool for fundamental research into plant stress signaling pathways and for the high-throughput screening of new crop varieties for enhanced climate resilience. While conventional methods for ABA detection, such as high-performance liquid chromatography-mass spectrometry (HPLC-MS), are highly accurate, they are also labor-intensive, expensive, and confined to centralized laboratories (Tang et al., 2024; Vrobel and Tarkowski, 2023), making them unsuitable for real-time, on-site analysis. ABA-specific scFvs (ABA-scFv) present a viable approach to support the development of low-cost, portable biosensors, such as lateral flow strips or electrochemical devices, for field-based applications (Pei et al., 2021; Müller et al., 2023). However, realizing this potential is hindered by a persistent obstacle: the difficulty of producing sufficient quantities of soluble, functional ABA-scFv in microbial hosts, a bottleneck that has severely limited its widespread adoption.

The prokaryotic host *Escherichia coli* is a primary system for recombinant protein production due to its rapid growth, low cost, and well-characterized genetics. Nevertheless, its cellular environment poses a formidable challenge for the correct folding of many complex eukaryotic proteins, including scFvs. The cytoplasm of *E. coli* is a reducing environment, which actively inhibits the formation of the conserved intramolecular disulfide bonds required to stabilize the intricate immunoglobulin fold of the VH and VL domains. Without these crucial bonds, the protein cannot achieve its native conformation, resulting in a non-functional product. This often leads to the misfolding and aggregation of the overexpressed polypeptide into dense, insoluble intracellular deposits known as inclusion bodies (Arana-Peña et al., 2021; Marco, 2025). Although active protein can sometimes be recovered from inclusion bodies through harsh chemical denaturation and subsequent refolding steps, this process is often inefficient and fails to yield sufficient quantities of active protein. To circumvent this, a common strategy involves fusing the scFv to a hefty, highly soluble protein tag. A more direct biological approach, however, is to directly address the root cause of misfolding by co-expressing molecular chaperones. These cellular machines are central to the protein quality control network, actively guiding nascent polypeptides toward their native state and preventing off-pathway aggregation. Chaperone systems like *DnaK/DnaJ/GrpE* and *GroEL/GroES* have proven highly effective in enhancing the soluble yields of a wide range of challenging recombinant proteins, including numerous scFvs of therapeutic interest (Rafeeq et al., 2021; Akbari et al., 2022; Cicardi et al., 2021).

Despite the well-documented success of chaperone-assisted folding for antibodies targeting large protein antigens, this powerful strategy remains critically under-explored for scFvs directed against small molecule haptens, such as ABA. A particularly promising but insufficiently investigated avenue is the potential synergy between different classes of chaperones that act at distinct stages of the folding process. It was hypothesized that a coordinated, multi-stage intervention—combining the early, co-translational stabilization provided by the ribosome-associated *Trigger Factor* (*TF*) with the subsequent, post-translational, ATP-dependent folding assistance of the *GroEL/GroES* complex—will be more effective than the action of either chaperone system in isolation (Hävermark et al., 2025; Roeselova et al., 2024). This represents a clear research gap in the field of small-molecule antibody production. Therefore, the central aim of this study is to systematically investigate the co-expression of the *TF* and *GroEL/ES* chaperone systems, both individually and in combination, to enhance the soluble expression and functional antigen-binding activity of ABA-scFv in the *E. coli* BL21 (DE3) strain. By identifying an optimized chaperone-assisted production strategy, we aim to establish a robust, efficient, and scalable platform for high-yield ABA-scFv manufacturing. Success in this endeavor will not only overcome a significant technical hurdle but will also accelerate the development of next-generation analytical tools for fundamental plant science and sustainable agriculture.

## 2 Materials and methods

### 2.1 Materials

The *E. coli* strain BL21 (DE3) was purchased from Sangon Biotech (China). The ABA-scFv gene sequence, referenced from Lu (2013), was synthesized by Sangon Biotech and cloned into the *pET30a* vector to create the expression plasmid *pET30a-ABA-scFv*. To establish the chaperone co-expression systems, BL21 (DE3) competent cells were first transformed with one of five molecular chaperone plasmids: *pG-KJE8*, *pGro7*, *pKJE7*, *pG-Tf2*, or *pTf16* (Takara, Japan), as detailed in Table 1. After selection and cultivation of these host strains, the *pET30a-ABA-scFv* plasmid was transformed into each, resulting in five distinct co-expression strains.

**TABLE 1:** Strains and plasmids used in this study.

Stain	Plasmid	Encoded chaperone(s)	Inducer(s)	Selection marker
Blank	pET30a	None	IPTG	Kanamycin
CK	pET30a-ABA-scFv	None	IPTG	Kanamycin
pG-KJE8	pET30a-ABA-scFv pG-KJE8	DnaK-DnaJ-GrpE and GroES-GroEL	IPTG& L-Arabinose and Tetracycline	Kanamycin Chloramphenicol
pGro7	pET30a-ABA-scFv pGro7	GroES-GroEL	IPTG& L-Arabinose	Kanamycin Chloramphenicol
pKJE7	pET30a-ABA-scFv pKJE7	DnaK-DnaJ-GrpE	IPTG& L-Arabinose	Kanamycin Chloramphenicol
pG-Tf2	pET30a-ABA-scFv pG-Tf2	GroES-GroEL and Tig	IPTG& Tetracycline	Kanamycin Chloramphenicol
pTf16	pET30a-ABA-scFv pTf16	Tig (Trigger Factor)	IPTG& L-Arabinose	Kanamycin Chloramphenicol

For systematic evaluation, an uninduced ABA-scFv strain (−IPTG) was included as a negative-expression control to subtract background signals from the host cells or assay reagents.

### 2.2 Quantification of ABA-scFv expression and solubility

The concentration of soluble scFv was determined by an indirect ELISA. This assay was specifically designed to quantify the yield of scFv variants presenting an accessible C-terminal His-tag. This approach ensures that the quantified protein is not only expressed but also correctly folded in a manner that allows for its detection and subsequent use in downstream immunoassays that rely on the His-tag for capture or detection. The use of highly specific monoclonal anti-His antibodies is a standard and reliable method for such quantification (Waugh, 2005; Gaberc-Porekar and Menart, 2001).

Firstly, 10 μL (OD600 diluted to 0.6) of each *E. coli* expression strain was inoculated into 10 mL of LB liquid medium containing 1 mM IPTG, 60 μg/L kanamycin (Kana), corresponding antibiotics for the molecular chaperone expression plasmids, and inducing agents (as shown in Table 1). The cultures were grown at 28 °C and 150 rpm until reaching the stationary phase, with four biological replicates for each treatment.

Upon reaching the stationary phase, cells were harvested by centrifugation. Biomass was measured directly as the wet weight of the bacterial pellet. To account for variations in cell density between cultures, the relative cell number was determined by quantifying the genomic copy number using absolute qPCR; this served as a normalization factor. This per-cell normalization is crucial for distinguishing the direct effects of chaperones on protein folding from their indirect effects on overall cell growth and biomass. The primers for the chromosomal reference were *E. coli*’s *dxs* monocopy gene (F: CGA​GAA​ACT​GGC​GAT​CCT​TA, R: CTT​CAT​CAA​GCG​GTT​TCA​CA) (Hernandez-Beltran et al., 2024). The qPCR cycling conditions were: 95 °C × 2.5 min; 50 cycles of (95 °C × 15 s, 55 °C × 30 s, 72 °C × 30 s) using a Real-time fluorescence quantitative PCR instrument (K9600, QuantReady, China), and the standard curve preparation followed the method by Shapiro et al. (2018).

The cells were centrifuged, and the wet weight of the bacterial pellet was measured. The bacterial protein extraction kit (CWBio, China) was used for cell lysis. The supernatant, containing the soluble expressed protein, was collected for analysis, while the pellet containing inclusion bodies was dissolved using the inclusion body solubilization buffer (Sangon Biotech, China) for separate quantification. The purified soluble protein from the supernatant was first verified for purity and correct molecular weight by SDS-PAGE. For further identity confirmation, a Western blot was performed. Proteins from the gel were transferred to a PVDF membrane, which was then blocked with 5% non-fat milk in TBST. The membrane was incubated overnight at 4 °C with a primary anti-His tag mouse monoclonal antibody (1:5,000 dilution, Sangon Biotech), followed by incubation for 1 h at room temperature with an HRP-conjugated goat anti-mouse IgG secondary antibody (1:10,000 dilution, Sangon Biotech). The signal was visualized using an ECL chemiluminescence kit. The protein content was measured using an enzyme-linked immunosorbent assay (ELISA) kit (Shanghai Yuanju Bio, China), and the absorbance was read at 450 nm using a microplate reader (Infinite E Plex, Tecan, Austria). All relevant operations followed the instructions provided with the respective kits. The final volumetric production of ABA-scFv was normalized against the initial culture volume and expressed in pmol per mL of the initial culture.

### 2.3 Optimization of ELISA parameters by checkerboard titration

The ABA conjugated antigen ABA-OVA was prepared according to Yan et al. (2022) using the dicyclohexylcarbodiimide (DCC) method. To prepare the ABA-OVA conjugate, 0.1 mmol of ABA was dissolved in 1.6 mL of N, N-dimethylformamide (DMF). Subsequently, 0.2 mL of 1.5 mol/L N-hydroxysuccinimide (NHS) in DMF was added, and the mixture was stirred for 15 min. Next, 2 mL of 0.1 mmol/L dicyclohexylcarbodiimide (DCC) in DMF was added to the solution, and the reaction was stirred overnight at room temperature. The mixture was centrifuged at 4,000 rpm for 10 min, and the resulting supernatant was collected and dialyzed against 0.1 M PBS for 3 days. Finally, the supernatant was freeze-dried. The resulting powder was re-dissolved in deionized water to a final concentration of 500 mg/L. Conjugation ratios were determined via intact isoform characterization of protein–hapten conjugates by LC-ESI-qTof mass spectrometry (Adamczyk et al., 1996) combined with spectral deconvolution using UniDec (Marty et al., 2015). OVA–ABA conjugates were desalted using 30 kDa ultrafiltration units (Briscale UF, Cobetter, China). Reversed-phase desalting was performed on a C4 column (ACQUITY UPLC Protein BEH C4, Waters, United States) using a binary gradient system, with solvent A consisting of 0.1% formic acid (FA) in water and solvent B consisting of 0.1% FA in acetonitrile. Proteins were eluted with a linear gradient from 5% to 95% B over 20 min at a flow rate of 0.3 mL/min. Intact protein masses were acquired on an ESI-QTOF instrument (6546 Q-TOF, Agilent Technologies, United States) under denaturing conditions, combined with spectral deconvolution using UniDec. Sample purity was validated by SDS-PAGE (Laemmli, 1970), which revealed a single dominant band at ∼45 kDa.The checkerboard ELISA procedure was conducted according to the method of Wang et al. (2021). For optimization, antigen coating dilutions of 1:50, 1:100, 1:200, 1:400, 1:800, 1:1,600, 1:3,200, and 1:6,400 (50 μL per well) were tested against antibody dilutions of 1:50–1:1,600 prepared from a 5 μg/mL stock using 0.1% PBS buffer containing 1% Tween-20 and 1 g/L gelatin (pH 7.5). In competitive binding wells, 50 μL of ABA at 100 ng/mL was added, and OD values of competitive binding and control wells were compared to identify the optimal antigen–antibody combination for each expression strain. Secondary antibody: HRP-conjugated Anti-HIS mouse monoclonal antibody, Sangon Biotech, cat#410002, dilution 1:2000. Subsequently, competitive ELISAs were performed under the optimized conditions. Serial ABA concentrations (0–1,000 ng/mL) were added to generate competitive binding curves, from which IC_50_ values were calculated by four-parameter logistic regression. All experiments were conducted in triplicate.

### 2.4 Analysis of ABA binding affinity for single-chain antibodies synthesized by various expression strains

For this analysis, the optimal coating antibody and antigen concentrations were used, as determined by the checkerboard titration described previously. The calculation method for IC_50_ refers to Han et al. (2024). Six concentration gradients of 1,000 ng, 500 ng, 250 ng, 125 ng, 62.5 ng, and 31.25 ng were used to establish standard curves for each treatment. The OD value at IC_50_ was calculated using Equation 1, and the ABA concentration at half-maximal inhibitory concentration (IC_50_) was determined from the standard curve to evaluate its sensitivity.

$$\text{OD at }{\text{IC}}_{50}=\text{OD value of control well}\times 50\%$$ (1)

### 2.5 Analysis of the recognition specificity of synthetic single-chain antibodies for expressed lines

The IC_50_ values for common plant interfering factors (IAA, GA3, L-lysine, and benzoic acid) were determined, using the optimal coating combination. Their cross-reactivity rates were then calculated with Equation 2 to evaluate the specificity of ABA recognition.

$$\text{Cross}-\text{reactivity rate }\left(\%\right)=\left({\text{IC}}_{50}\;\left(\text{ABA}\right)\;/\;{\text{IC}}_{50}\;\left(\text{interfering factor}\right)\right)\times 100$$ (2)

### 2.6 FT-IR analysis

The purified ABA-scFv samples were mixed with KBr (w/w = 1/140), then vacuum freeze-dried and pressed into thin pellets. The infrared spectrum was recorded between 4,000 and 450 cm^-1^ (Spectrum Two; PerkinElmer, Waltham, MA, United States). Each spectrum was obtained using a resolution of 1 cm^-1^. This analysis was performed on proteins purified from three independent biological replicates, and representative spectra were presented. The spectrum absorption in the amide I region (1,600–1700 cm^-1^) was analyzed using the Peakfit (Sea Solve Software Inc., Shanghai, China) (Vanga et al., 2020). The secondary structure of the ABAs-cFv protein was predicted using Phyre2 (https://www.sbg.bio.ic.ac.uk/phyre2/html) with default parameters (Kelley et al., 2015). The full-length amino-acid sequence of the expressed protein (VL–linker–VH; 239 aa, 25.6 kDa), including the C-terminal His-tag, was used for this analysis to ensure direct comparability with experimental data.

### 2.7 Analysis of CD spectra

The concentrations of the purified ABA-scFv samples were determined using a Nanodrop (ThermoFisher, United States) and then diluted to 0.1 mg/mL with PBS containing 0, 25, 50, and 100 ng/μL. All measurements were performed on samples from three independent biological replicates. Parameters of the circular dichroism spectrometer (Chirascan, Applied Photophysics, England) were adjusted (wavelength range: 190–250 nm, optical path difference: 1 mm). The zero point and reference background (the blank solvent) were calibrated, and a special cuvette was used to avoid bubbles. A stable temperature (usually 20–25 °C) was maintained during data acquisition, an appropriate scanning speed was set to obtain a clear spectrum, and absorption differences between the sample and reference spectra were recorded. Baseline correction and smoothing denoising were performed during data processing, and the secondary structure and conformational changes were analyzed by curve fitting or software CDNN (Applied Photophysics).

### 2.8 Data processing and statistical analysis

Analysis of variance (ANOVA) and multiple comparisons (Duncan’s method) were performed using SPSS (IBM, United States). Relevant charts and graphs were created using Origin (OriginLab, United States).

## 3 Results

### 3.1 The effect of different molecular chaperones on the synthesis of ABA-scFv

**FIGURE 1:**
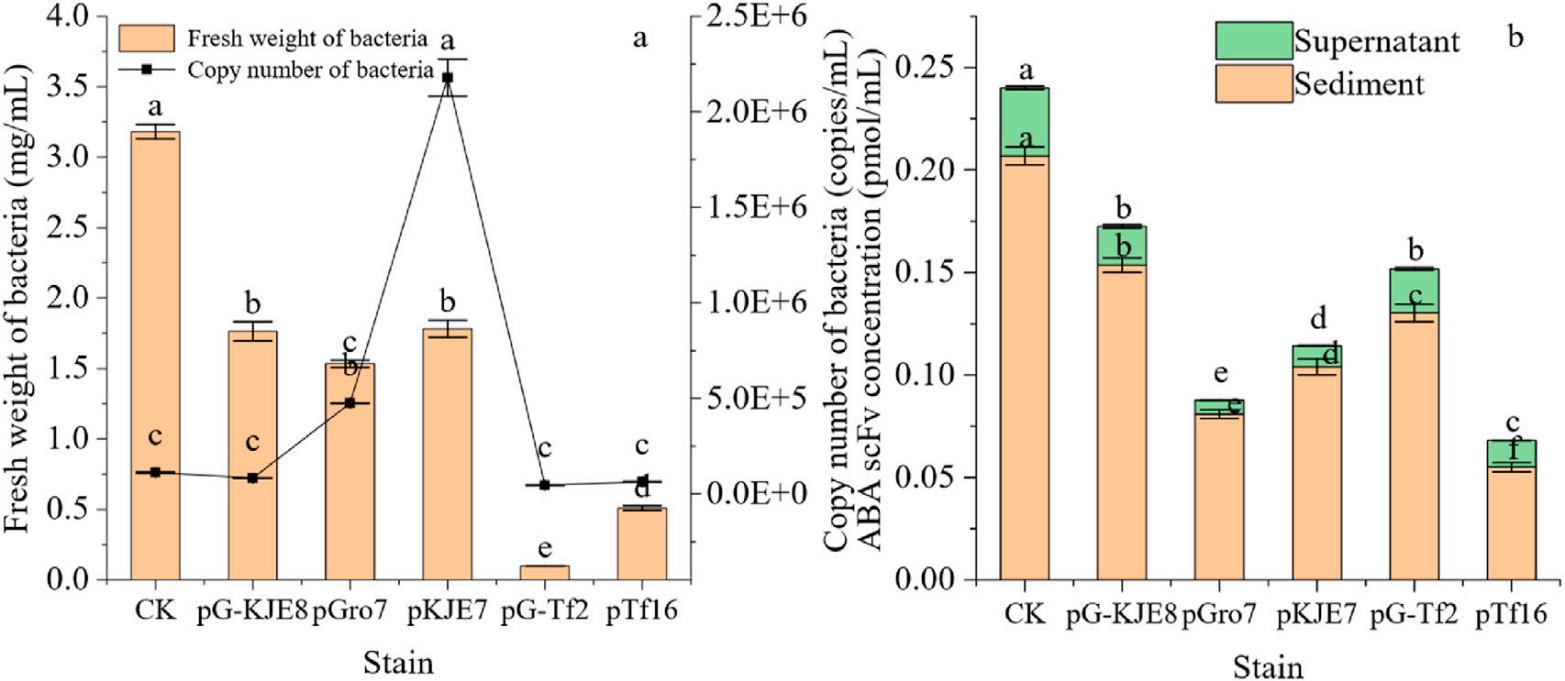
Effects of Different Molecular Chaperones on the Expression of *ABA-scFv* in *E. coli*. **(a)** Cell biomass (fresh weight) and genomic copy number at the stationary phase. **(b)** ABA-scFv in soluble (supernatant) vs. insoluble (pellet) fractions quantified via His-tag ELISA after Ni-NTA purification. Bars show mean ± SE (n = 3). Lowercase letters indicate significant differences (p < 0.05) among treatments, based on one-way ANOVA followed by Duncan’s multiple range test. CK: *ABA-scFv* expression strain induced with IPTG but without chaperone plasmid (baseline control). An empty vector control and an uninduced control (−IPTG) were also analyzed, which showed no or negligible protein expression, respectively (data not shown).

The growth and synthesis of single-chain antibodies in different molecular chaperone strains are shown in Figure 1. After co-expression with molecular chaperones, only the *pGro7* and *pKJE7* treatments significantly increased the bacterial cell number, while other treatments showed no significant difference compared to the control. However, in terms of biomass, co-expression of molecular chaperones reduced biomass for all strains, with the *pG-TF2* treatment showing the most notable decrease. The *pGro7* and *pKJE7* strains exhibited higher cell numbers than the control. However, their final biomass was approximately 50% lower, indicating that increased cell proliferation did not correlate with higher protein synthesis under these conditions.

In all treatments, the control showed relatively higher protein synthesis efficiency. When interpreting the volumetric yields, it is crucial to consider the significant variations in cell number and biomass across the treatments, which indicate differing levels of metabolic load imposed by the chaperone systems. Regarding the synthesis of single-chain antibodies, the quantifiable yield of *ABA-scFv*, as determined by His-tag ELISA, was lower in all chaperone co-expression systems compared to the control. In terms of expression activity, although the majority of the expressed scFv remained in the insoluble fraction for all strains, co-expression with *pTf16* significantly increased the proportion of detectable soluble scFv to 19.65%, compared to 14.20% in the control strain (Figure 1b), indicating that *pTf16* was the most effective in assisting protein folding. SDS-PAGE and Western blot analysis of this purified soluble protein confirmed the presence of a single, distinct band at the expected molecular weight of ∼26 kDa, with no smaller fragments indicative of protein degradation observed (Supplementary Figure S3).

### 3.2 The effect of different molecular chaperones on the binding affinity of ABA-scFv

Prior to checkerboard titration analysis, the integrity of the OVA–ABA conjugates was validated by intact MS deconvolution (Supplementary Figure S2b), which showed a clear ladder of conjugated species separated by Δm ≈ 246 Da. The distribution centered at k = 6–10 with an average conjugation number of 8.21, indicating high conjugation efficiency (99.16%) with negligible unmodified OVA (<1%). In addition, SDS-PAGE analysis revealed a single dominant band at ∼45 kDa (Supplementary Figure S1), confirming the purity and integrity of the conjugates. These results demonstrated that the OVA–ABA conjugates were suitable for immunoassay development.

We therefore proceeded to screen the optimal antigen coating concentration and antibody dosage for each variant in the checkerboard titration assay. It is important to note that this individual optimization was a critical step in our experimental design. As different molecular chaperone systems can distinctly influence the folding efficiency, stability, and binding characteristics of the expressed scFv, establishing a unique, optimized assay condition for each is essential. The objective of this study was not to compare the absolute binding affinities of the scFv variants under a single, potentially suboptimal condition for all. Instead, the goal was to evaluate the maximum functional performance of each ‘chaperone-antibody system’ to determine its suitability for specific applications. The experimental results indicate that, in each treatment, higher coating concentrations or antibody dosages resulted in higher OD values in the control wells and, correspondingly, higher OD values in the competitive binding wells. This result suggests that within the working concentration range (100 ng/mL ABA), excessively high concentrations of coated antigens and antibodies weaken the competitive binding between free ABA (target ABA) and the antibody, thereby reducing sensitivity. On the other hand, excessively low concentrations of coated antigens and antibodies are insufficient to provide enough binding sites for the primary antibody and enzyme-labeled secondary antibody. Therefore, appropriately diluted coating antigens and antibodies are crucial for maintaining a relatively low OD value in the competitive binding wells while ensuring a significant difference from the control wells, thus improving antibody detection sensitivity. Finally, we selected the following conditions: For the control (CK): antigen coating dilution 100 times (50 mg/L), antibody dilution 800 times (6.25 μg/L). For *pG-KJE8*: antigen coating dilution 1,600 times (3.125 mg/L), antibody dilution 100 times (0.05 mg/L). For *pGro7*: antigen coating dilution 1,600 times (3.125 mg/L), antibody dilution 100 times (0.05 mg/L). For *pKJE7*: antigen coating dilution 3,200 times (1.5625 mg/L), antibody dilution 800 times (6.25 μg/L). For *pG-Tf2*: antigen coating dilution 100 times (50 mg/L), antibody dilution 50 times (0.1 mg/L). For *pTf16*: antigen coating dilution 1,600 times (3.125 mg/L), antibody dilution 800 times (6.25 μg/L).

**FIGURE 2:**
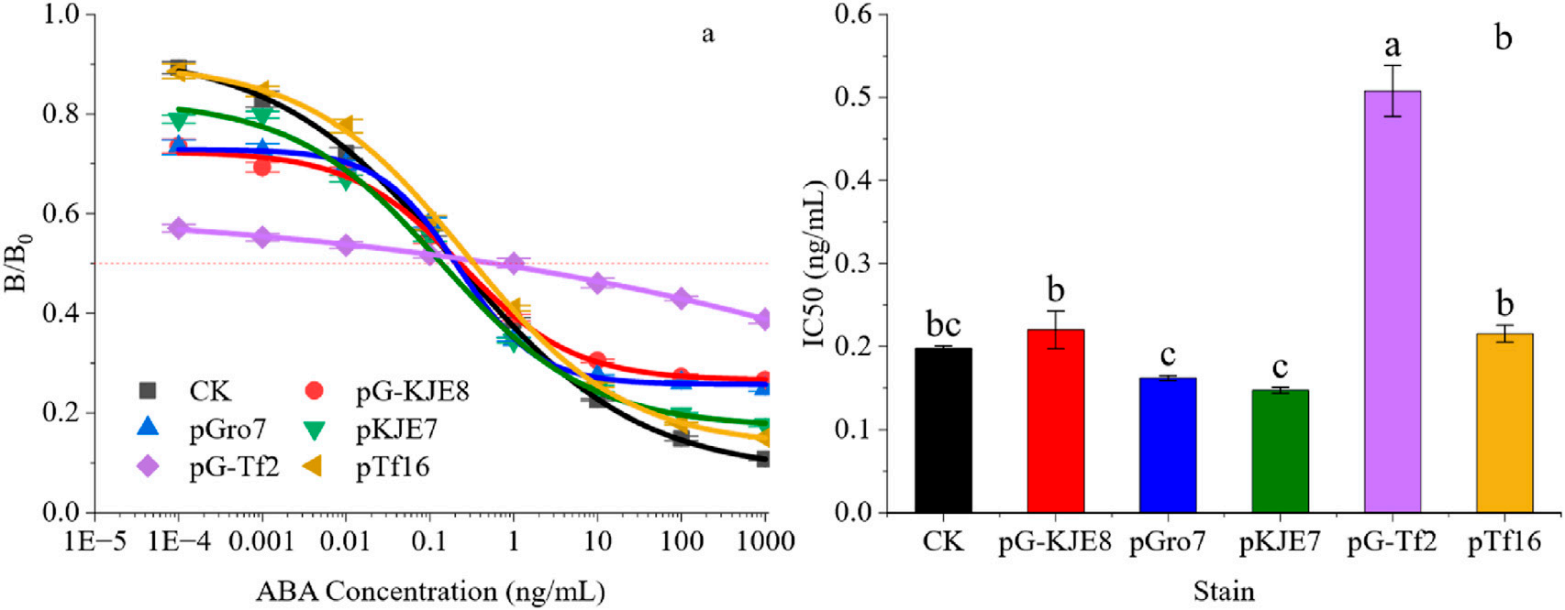
Different Molecular Chaperone Co-expression Systems Affect ABA-scFv Folding and Binding Abilities. **(a)** Fitting of the standard curve for single-chain antibody ABA synthesis. **(b)** IC_50_ Differences in Different Fitting Curves. Bars show mean ± SE. Different letters above bars indicate significant differences by one-way ANOVA followed by Duncan’s multiple range test (p < 0.05).

To further analyze the binding affinity of each antibody, the IC_50_ of the antibodies was analyzed at the optimal antigen coating concentration and antibody dosage for each treatment, as shown in Figure 2 The standard curves of all treatments co-expressing and synthesizing ABA single-chain antibodies could be successfully fitted (R^2^ ≥ 0.99). In terms of sensitivity and linear range (measured as the IC_20_ to IC_80_ interval), the antibodies exhibited different properties after assisted folding by various molecular chaperones.

The *pKJE7*-assisted scFv displayed the highest sensitivity, with an IC50 value 25.37% lower than that of the control; however, this was accompanied by a narrower linear detection range (IC_20_-IC_80_). In contrast, the *pTf16* treatment significantly extended the detection linear range of the antibodies, but there was no significant improvement in sensitivity compared to the CK.

### 3.3 The effect of different molecular chaperones on the specificity of ABA-scFv recognition

The cross-reactivity rates of different ABA single-chain antibodies were analyzed to verify the recognition specificity of ABA single-chain antibodies under different molecular chaperone-assisted syntheses (Table 2). The results showed that, except for the *pG-Tf2* treatment, all molecular chaperone treatments significantly reduced the non-specific binding of ABA antibodies, particularly addressing the high cross-reactivity with indole-3-acetic acid (IAA) observed in the control. The co-expression with the *pTf16* molecular chaperone resulted in the lowest cross-reactivity rate of ABA single-chain antibodies. Notably, the *pG-Tf2* treatment significantly increased the cross-reactivity rates of the single-chain antibody with various substances, indicating that this treatment adversely affected the specificity of the ABA-scFv.

**TABLE 2:** Cross-reactivity of ABA-scFv synthesis for each treatment.

Competitive antigen	L-lysine		Benzoic acid		Heteroauxin		Gibberellin	
	IC_50_ (ng/mL)	Cross-reactivity (%)	IC_50_ (ng/mL)	Cross-reactivity (%)	IC_50_ (ng/mL)	Cross-reactivity (%)	IC_50_ (ng/mL)	Cross-reactivity (%)
CK	225.87 ± 7.82	0.077	2,750.33 ± 121.16	<0.001	0.122 ± 0.003	164.10	64.64 ± 9.01	0.3048
*pG-KJE8*	529.37 ± 21.97	0.040	>10,000	<0.001	62.058 ± 1.38	0.3451	44.01 ± 1.470	0.4811
*pGro7*	186.57 ± 22.99	0.090	193.93 ± 47.11	0.0864	158.35 ± 3.88	0.1058	147 ± 22.61	0.1032
*pKJE7*	>10,000	<0.001	>10,000	<0.001	1,028.56 ± 32.82	0.0144	3,239.22 ± 393.47	0.0046
*pG-Tf2*	92.85 ± 2.30	0.5357	1.049 ± 0.020	47.45	33.93 ± 0.70	1.4659	59.09 ± 3.70	0.8417
*pTf16*	>10,000	<0.001	>10,000	<0.001	>10,000	<0.001	>10,000	<0.001

### 3.4 Secondary structure analysis of ABA-scFv

**FIGURE 3:**
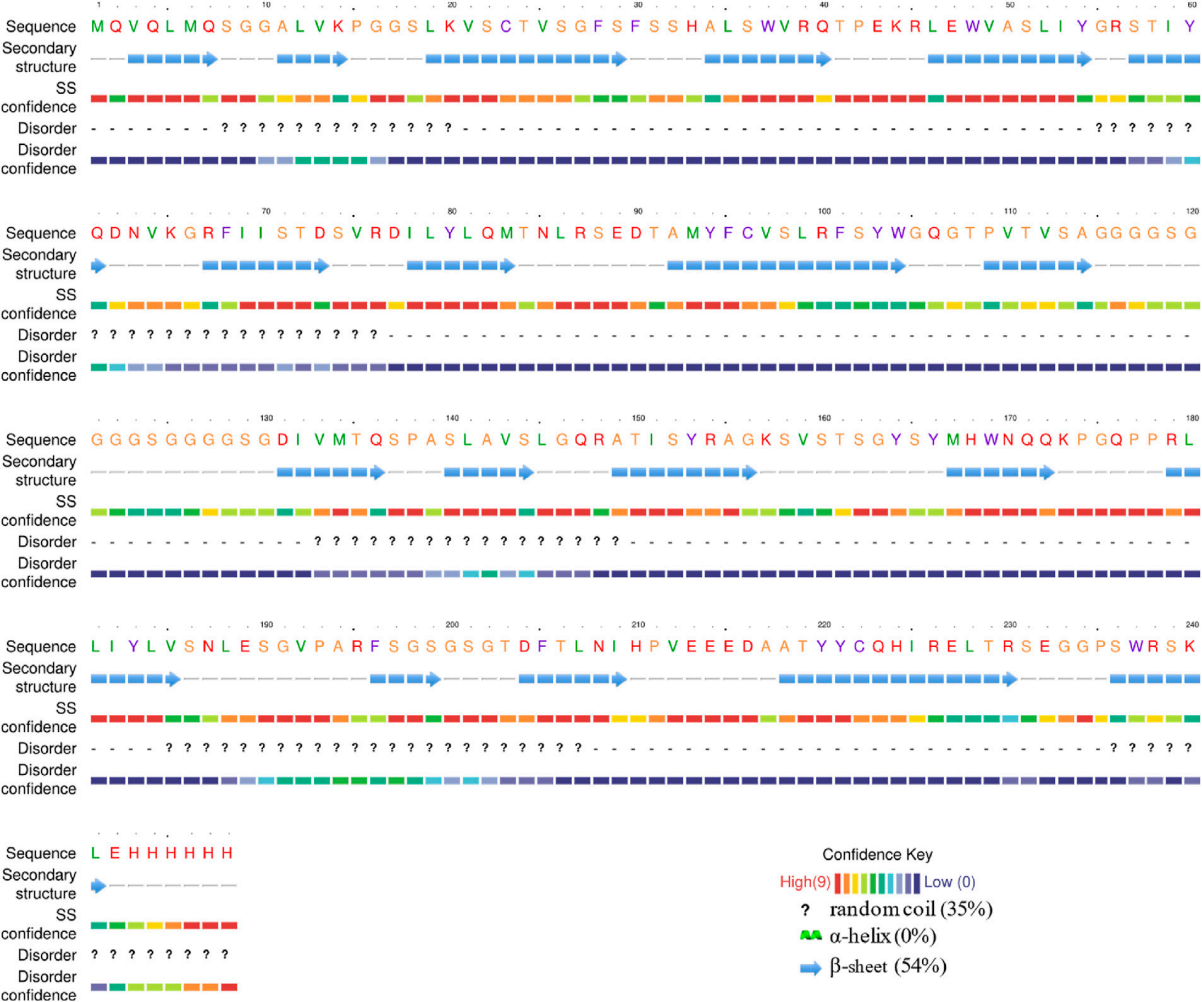
Secondary structure prediction of ABA-scFv.

The impact of different molecular chaperones on the secondary structure of the ABA single-chain antibody was investigated using ATR-FTIR spectroscopy, with percentages of each structure quantified by fitting the amide I band. For comparison, the *in silico* structure prediction for the ABA-scFv indicated a conformation redominantly composed of β-sheet (54%) and disordered regions (35%), with a notable absence of α-helices (0%) (Figure 3).

**FIGURE 4:**
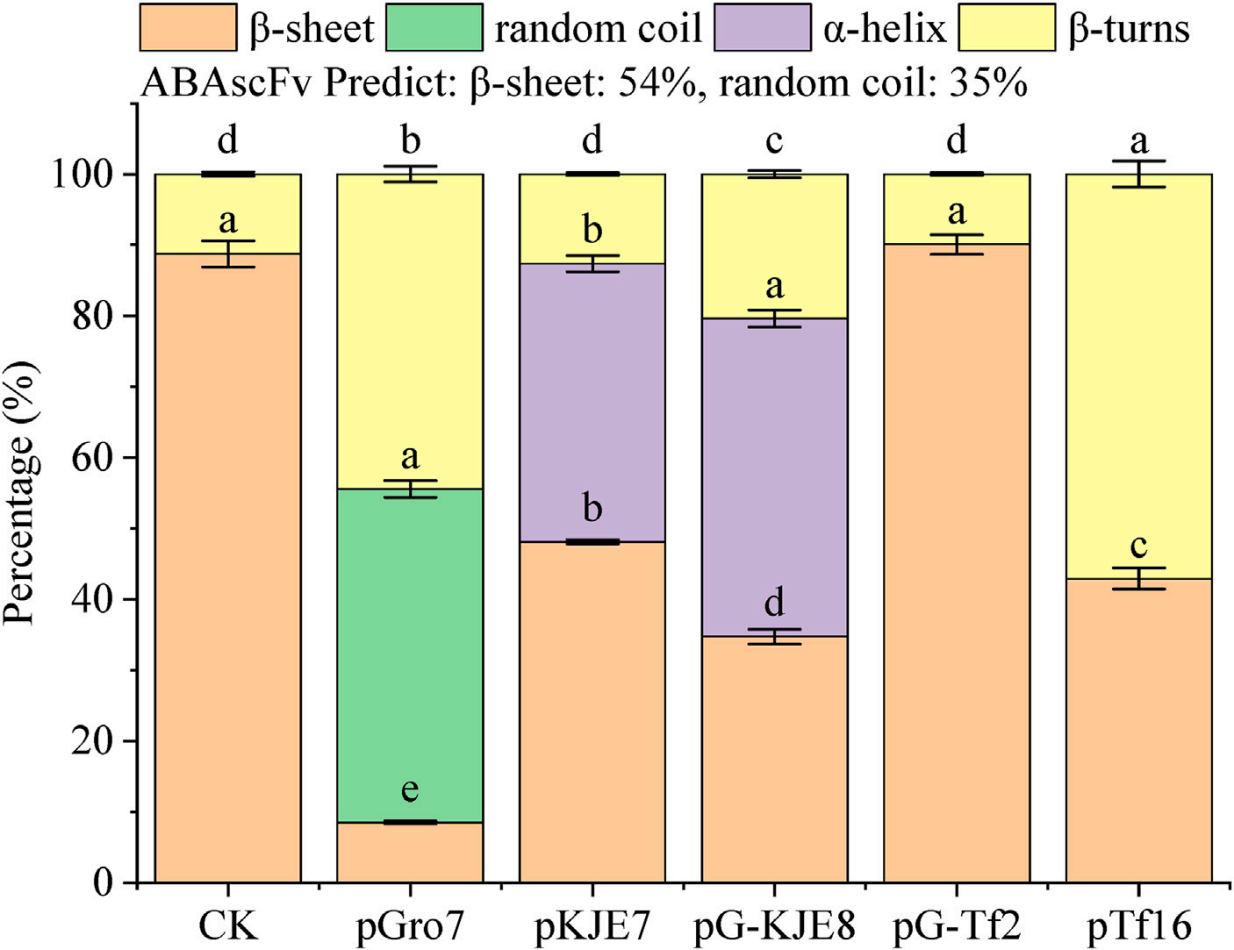
Secondary structure percentages. Bars represent mean ± SE. Lowercase letters indicate significant differences (p < 0.05) among treatments within each secondary structure (one-way ANOVA + Duncan’s test).

Our experimental findings revealed significant structural alterations across the treatments (Figure 4). A key finding was in the β-sheet content, where the *pKJE7* treatment produced a structure with 48.2% β-sheet, the most similar proportion to the predicted value of 54%. The pTf16 treatment, with a β-sheet content of 43.4%, ranked as the second most similar to the prediction. Another critical finding was in the α-helix content; the pTf16 system produced an scFv with 0% α-helix, perfectly matching the prediction, whereas the *pKJE7* treatment resulted in 12.5% α-helix content, a notable deviation from the theoretical model.

Based on these findings, while no single chaperone perfectly replicated the predicted structure, *pKJE7* was most effective in forming a native-like β-sheet core, whereas *pTf16* was most effective in preventing the formation of non-native α-helical structures.

### 3.5 The influence of different concentrations of ABA on the secondary structures of single-chain antibodies under different treatments

**FIGURE 5:**
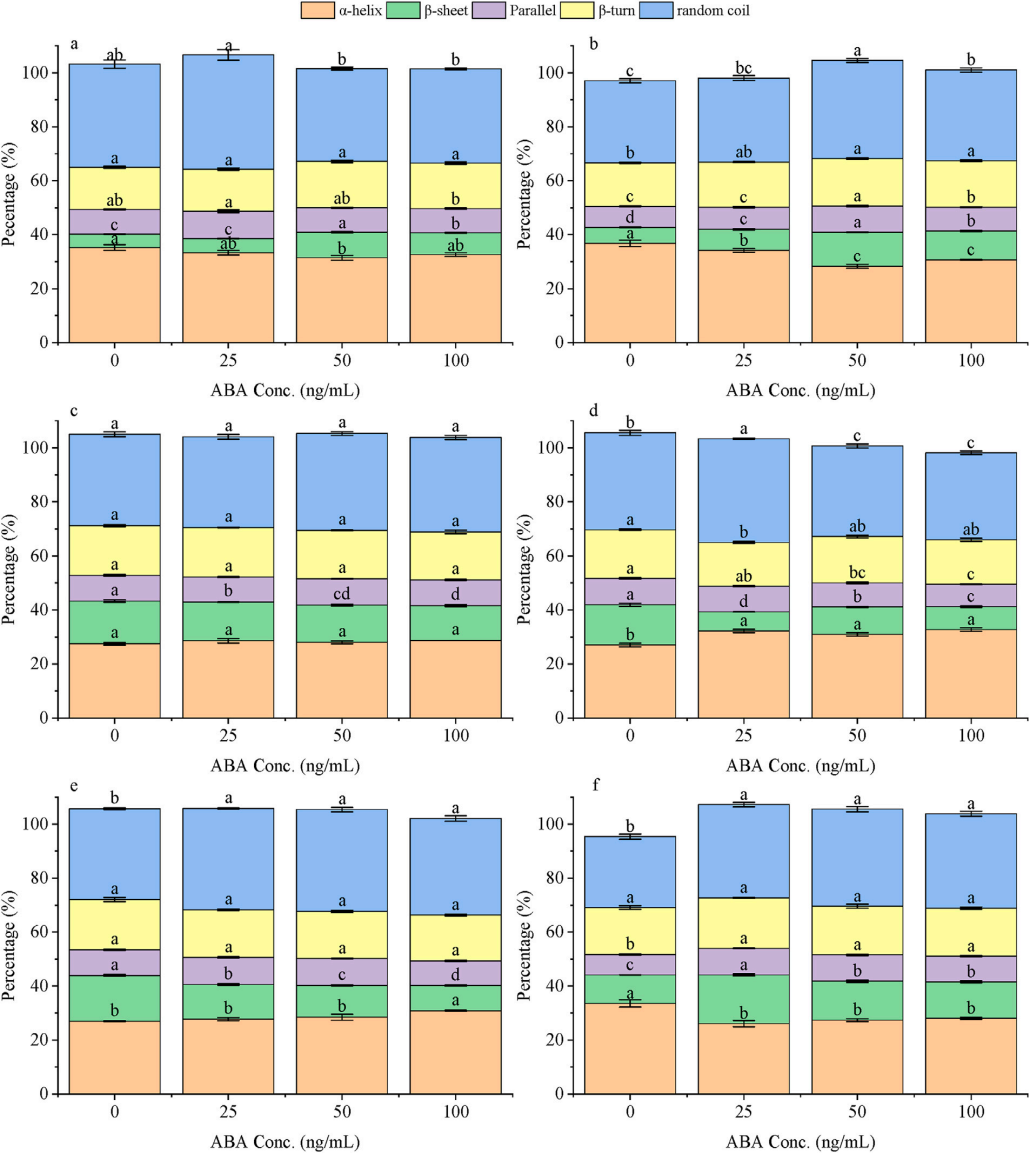
The differences in the secondary structure of ABA-scFv determined by circular dichroism spectroscopy under different ABA concentrations (0, 25, 50, 100 ng/μL) treatment with various molecular chaperones. Structural conformational alterations of ABA-scFv under ABA concentration gradients with CK **(a)**, *pGro7* **(b)**, *pKJE7* **(c)**, *pG-KJE8* **(d)**, *pG-Tf2* **(e)** and *pTf16* **(f)** treatment. Bars represent mean ± SE. Lowercase letters indicate significant differences (p < 0.05) among treatments within each secondary structure (one-way ANOVA + Duncan’s test).

To gain mechanistic insight into the antigen recognition event, circular dichroism spectroscopy was used to monitor the conformational response of each scFv variant upon binding to ABA (Figure 5). The data revealed two fundamentally different modes of interaction depending on the chaperone system used.

First, the scFv produced by the *pKJE7*, *pG-KJE8*, and *pTf16* systems exhibited a conformationally rigid response to ABA recognition. Their secondary structures remained stable across all tested ABA concentrations. For instance, the β-sheet content of the pKJE7 variant only fluctuated between 15.8% and 16.1%. This structural stability upon antigen binding suggests these chaperones produce scFv that exists in a pre-formed, ‘binding-ready’ state, consistent with what is expected for a lock-and-key mechanism.

In stark contrast, the scFv from the control (CK), *pGro7*, and *pG-Tf2* systems displayed a conformationally flexible response, undergoing significant structural changes upon interaction with ABA. This was most evident in the *pG-Tf2* variant, which showed a substantial decrease in β-sheet content from a baseline of 16.1%–11.1%. This structural rearrangement upon antigen binding suggests an ‘induced fit’ recognition mechanism, where the antibody must alter its conformation to achieve optimal binding.

## 4 Discussion

This study demonstrates that the strategic co-expression of molecular chaperones, notably Trigger Factor, can help address solubility and functionality challenges in ABA-scFv production. Our findings indicate a relationship between the chaperone-assisted attainment of a native-like secondary structure and the antibody’s binding performance.

A notable and somewhat counterintuitive finding was that while some chaperone systems improved the soluble fraction, all co-expression treatments resulted in a lower total protein yield and reduced host cell biomass compared to the control. This highlights a trade-off between production quantity and functional quality. This outcome arises from a combination of physiological factors. First, the overexpression of chaperones imposes a significant metabolic burden, consuming substantial cellular resources (Hoffmann and Rinas, 2004; Glick, 1995). Second, interference between the different induction systems is a probable cause; the simultaneous activation of the arabinose/tetracycline-regulated chaperone plasmids and the IPTG-inducible scFv expression plasmid can lead to transcriptional and translational interference, creating a bottleneck that limits optimal target protein expression (Saïda et al., 2006).

To evaluate each system, we optimized assay conditions for each treatment. Therefore, the goal of this comparison was not a rigid determination of intrinsic molecular affinity (K_d_), but a more practical, system-level, application-oriented assessment. The objective was to determine which overall technical solution—encompassing the chaperone system, expression, and final assay—provides the most suitable performance for a given application, such as achieving maximum sensitivity (the lowest IC_50_ value) or the widest quantitative range (the broadest IC_20_-IC_80_).

An important observation is the intricate relationship between subtle secondary structure variations and the distinct functional specializations of the ABA-scFv. Our data reveal a functional trade-off that can be directly linked to chaperone-induced conformational differences.

The *pKJE7* system, which produced an scFv with the highest sensitivity (lowest IC_50_), also yielded a secondary structure whose β-sheet content (48.2%) was the most similar to the *in silico* prediction (54%). This strong correlation suggests that achieving a native-like β-sheet core is important for forming a high-affinity binding pocket. This outcome can be attributed to the powerful mechanism of the *DnaK-DnaJ-GrpE* system encoded by *pKJE7*. As a central machine in the cell’s heat shock response, it functions by recognizing and binding to exposed hydrophobic patches on misfolded proteins, using ATP hydrolysis to unfold and release them, providing a “second chance” at proper folding (Straus et al., 1990). This potent refolding capacity likely explains its success in achieving a near-perfect β-sheet core, which correlates with maximum sensitivity.

Conversely, the *pTf16* system, which delivered superior specificity and a broader dynamic range, produced a structure that was unique in another critical aspect: it contained 0% α-helix, perfectly matching the prediction. This structural ‘purity’ may contribute to the observed specificity. *Trigger Factor*’s unique mechanism can explain this. As the first chaperone to encounter the nascent polypeptide as it exits the ribosome, it acts to prevent misfolding from the outset, shielding hydrophobic regions and guiding the protein along a productive folding pathway (O’Brien et al., 2012; Valent et al., 1995). This preventative, co-translational action likely accounts for the formation of a structurally ‘pure’ conformation, free of non-native α-helices, which in turn creates a more defined and less promiscuous binding interface that effectively rejects interfering molecules.

Furthermore, our analysis of the scFv’s conformational response to ABA provides deeper mechanistic insight into why these top-performing chaperone-assisted variants are superior. As shown by the CD spectroscopy data, the scFv from the *pKJE7* and *pTf16* systems exhibit a conformationally rigid response, maintaining their structural integrity upon antigen binding. This is consistent with a classic “lock-and-key” recognition mechanism, where the protein exists in a pre-formed, ‘binding-ready’ state that is directly complementary to the antigen (Csermely et al., 2010). For an immunoassay reagent, this structural rigidity may improve binding kinetics and assay consistency.

In contrast, the unstable variants from the control (CK) and *pGro7* systems displayed an “induced fit” mechanism, requiring structural rearrangement to bind the antigen. This model posits that the initial interaction triggers a conformational change in the protein to optimize the binding interface (Csermely et al., 2010). While a valid biological process, this flexibility can be a liability for a diagnostic tool, potentially leading to slower binding and a reduced net affinity, as the energetic cost of rearrangement consumes a portion of the binding energy (Schmitt et al., 2009). Therefore, the success of the *pKJE7* and *pTf16* systems lies not only in producing a structure with the correct secondary components but in producing a structure that employs a more efficient and robust antigen recognition mechanism.

The failure of the *pG-Tf2* system provides further evidence for the importance of matching the proper chaperone to the target protein. This system combines Trigger Factor with the *GroEL-GroES* machine, providing a protected folding environment. *GroEL-GroES* forms a cage-like structure that encapsulates a single unfolded polypeptide, isolating it from the crowded cytoplasm. Inside this “Anfinsen cage,” fueled by ATP hydrolysis, the protein can fold unimpeded (Kerner et al., 2005). The collapse of the *pG-Tf2* scFv, confirmed by its high cross-reactivity and explained by its elevated β-sheet content from our CD spectroscopy data, suggests a potential conflict between these two systems. The early binding of Trigger Factor may have interfered with the proper entry of the scFv into the *GroEL* cage, leading to a frustrated folding pathway that directed the protein into a non-native, aggregated state (Zhu et al., 2016; Fändrich, 2007).

Furthermore, our analysis of the antibody’s structural stability in the presence of its antigen revealed that the scFv produced with the *pTf16* and *pKJE7* systems maintained a stable secondary structure even at high ABA concentrations, a desirable trait for a desirable trait for an immunoassay reagent. This suggests that the choice of chaperone not only influences the initial fold but also the conformational resilience of the final antibody product.

A central aspect of this study’s platform was the quantification of scFv based on the ELISA detection of the C-terminal His-tag. We acknowledge that this method quantifies the yield of scFv with an accessible His-tag, which may not represent the absolute total yield of the expressed protein, as a fraction might misfold in a way that buries the tag. However, this was a deliberate and central aspect of our experimental design. For the intended downstream application of this scFv in immunoassays—where the His-tag is essential for capture or secondary antibody recognition—any scFv variant with an inaccessible tag is functionally irrelevant. Therefore, our quantification strategy provides a more practical and meaningful measure of the ‘useful’ or ‘application-ready’ scFv yield, rather than the absolute translational output, concurrently assessing both expression quantity and conformational quality.

This practical approach allowed us to determine that the optimized scFv produced with the pTf16 system demonstrates the high specificity and reliability required for immunoassays, thereby fulfilling the central objective of this study. Consequently, this work provides a basis for a low-cost platform for producing tailored ABA-scFv antibodies, providing insights into how different chaperone systems affect function. Building upon this, our future work will focus on developing high-sensitivity electrochemical immunosensors and user-friendly lateral flow assays. A critical component of this next stage will involve not only rigorous validation against the gold-standard HPLC-MS method but also more in-depth biophysical characterization of the lead antibody candidate, including binding kinetics and thermal stability analyses. This will bridge the gap from a functional screening platform to a fully characterized diagnostic tool ready for the field.

## 5 Conclusion

In conclusion, Trigger Factor (*Tig*), when expressed alone from the *pTf16* plasmid, was the most effective chaperone for producing a soluble, highly specific ABA-scFv with a secondary structure most consistent with the predicted native fold in *E. coli*. While combinations with other chaperone systems failed to provide synergistic benefits, some, like the *DnaK-DnaJ-GrpE* system from *pKJE7*, offered higher sensitivity at the cost of specificity. Therefore, these results suggest that chaperone selection can be tailored to specific downstream applications: the *pKJE7* system is optimal for immunoassays requiring maximum sensitivity, whereas the *pTf16* system is superior for those demanding high specificity and a broad dynamic range. By establishing and functionally validating these chaperone-optimized production strategies, this research provides a practical basis for developing low-cost ABA immunoassays for agricultural applications.

## Data Availability

The original contributions presented in the study are included in the article/Supplementary Material, further inquiries can be directed to the corresponding author.
