# Supplementary material for: Molecular chaperone effects on recombinant yield and binding characteristics of an ABA-specific scFv in Escherichia coli
Source: Front Bioeng Biotechnol. 2025 Oct 9;13:1643833. doi: 10.3389/fbioe.2025.1643833 (PMC12547695; doi:10.3389/fbioe.2025.1643833)
Supplement: Supplementary file 1 [file Supplementaryfile1.docx]

**Table S1 Results from screening optimal concentrations in ABA scFv antibodies with molecular chaperones and coated antigen**

| Molecular chaperone treatments | Dilution of antigen | Dilution of antibody | | | | | | | | | | | |
| --- | --- | --- | --- | --- | --- | --- | --- | --- | --- | --- | --- | --- | --- |
|  |  | 1:50 | | 1:100 | | 1:200 | | 1:400 | | 1:800 | | 1:1600 | |
|  |  | C | I | C | I | C | I | C | I | C | I | C | I |
| CK | 1:50 | 2.076 | 1.084 | 2.073 | 1.262 | 1.617 | 0.901 | 1.447 | 0.523 | 1.370 | 0.382 | 1.199 | 0.681 |
|  | 1:100 | 2.069 | 1.076 | 2.075 | 1.064 | 1.479 | 0.861 | 1.508 | 0.668 | 1.495 | 0.163 | 1.004 | 0.591 |
|  | 1:200 | 1.869 | 1.071 | 1.865 | 0.962 | 1.288 | 0.759 | 1.625 | 0.765 | 0.956 | 0.258 | 0.973 | 0.566 |
|  | 1:400 | 1.672 | 0.979 | 1.561 | 0.869 | 1.076 | 0.66 | 1.357 | 0.655 | 0.772 | 0.257 | 0.757 | 0.471 |
|  | 1:800 | 1.463 | 0.875 | 1.354 | 0.753 | 0.958 | 0.505 | 1.191 | 0.566 | 0.630 | 0.159 | 0.673 | 0.346 |
|  | 1:1600 | 1.277 | 0.724 | 1.061 | 0.667 | 0.705 | 0.490 | 0.929 | 0.393 | 0.484 | 0.185 | 0.508 | 0.222 |
|  | 1:3200 | 1.080 | 0.685 | 0.863 | 0.568 | 0.632 | 0.426 | 0.837 | 0.310 | 0.325 | 0.175 | 0.422 | 0.272 |
|  | 1:6400 | 0.903 | 0.566 | 0.505 | 0.258 | 0.551 | 0.382 | 0.644 | 0.283 | 0.227 | 0.174 | 0.393 | 0.285 |
| pG-KJE8 | 1:50 | 2.241 | 1.320 | 2.273 | 1.203 | 1.889 | 1.274 | 1.286 | 0.722 | 1.287 | 0.751 | 1.150 | 0.620 |
|  | 1:100 | 2.227 | 1.28 | 2.639 | 1.181 | 1.665 | 1.205 | 1.218 | 0.572 | 1.215 | 0.716 | 1.151 | 0.504 |
|  | 1:200 | 1.825 | 1.252 | 2.759 | 1.186 | 1.676 | 1.019 | 1.223 | 0.449 | 1.166 | 0.621 | 0.857 | 0.479 |
|  | 1:400 | 1.726 | 1.234 | 2.674 | 1.166 | 1.594 | 0.741 | 1.023 | 0.375 | 0.967 | 0.551 | 0.752 | 0.473 |
|  | 1:800 | 1.377 | 1.19 | 2.746 | 0.909 | 1.299 | 0.617 | 0.711 | 0.278 | 0.762 | 0.562 | 0.575 | 0.369 |
|  | 1:1600 | 1.449 | 1.224 | 2.837 | 0.762 | 0.910 | 0.392 | 0.607 | 0.244 | 0.678 | 0.346 | 0.373 | 0.199 |
|  | 1:3200 | 1.236 | 0.799 | 2.461 | 0.783 | 0.910 | 0.397 | 0.551 | 0.240 | 0.403 | 0.245 | 0.189 | 0.129 |
|  | 1:6400 | 1.449 | 0.669 | 2.242 | 0.671 | 0.851 | 0.232 | 0.376 | 0.227 | 0.286 | 0.188 | 0.190 | 0.122 |
| pGro7 | 1:50 | 2.214 | 1.459 | 1.832 | 1.286 | 1.331 | 0.738 | 1.449 | 0.807 | 1.268 | 0.696 | 0.841 | 0.324 |
|  | 1:100 | 2.404 | 1.558 | 1.734 | 1.296 | 1.373 | 0.684 | 1.610 | 0.668 | 1.237 | 0.659 | 0.821 | 0.303 |
|  | 1:200 | 2.269 | 1.587 | 1.317 | 1.335 | 1.292 | 0.947 | 1.601 | 0.602 | 0.961 | 0.714 | 0.541 | 0.294 |
|  | 1:400 | 1.772 | 1.624 | 1.437 | 0.632 | 1.300 | 0.942 | 1.367 | 0.534 | 0.765 | 0.688 | 0.430 | 0.267 |
|  | 1:800 | 1.840 | 1.247 | 1.585 | 0.599 | 1.226 | 0.579 | 1.216 | 0.497 | 0.623 | 0.550 | 0.380 | 0.240 |
|  | 1:1600 | 1.202 | 1.018 | 1.591 | 0.416 | 1.022 | 0.424 | 0.924 | 0.429 | 0.471 | 0.392 | 0.310 | 0.299 |
|  | 1:3200 | 1.188 | 0.814 | 1.437 | 0.412 | 0.925 | 0.532 | 0.846 | 0.414 | 0.363 | 0.215 | 0.198 | 0.172 |
|  | 1:6400 | 1.026 | 0.618 | 1.253 | 0.399 | 0.872 | 0.280 | 0.653 | 0.345 | 0.254 | 0.295 | 0.220 | 0.138 |
| pKJE7 | 1:50 | 2.42 | 1.264 | 2.219 | 1.295 | 1.734 | 1.180 | 1.268 | 1.022 | 0.526 | 0.516 | 0.262 | 0.165 |
|  | 1:100 | 2.228 | 1.201 | 1.916 | 1.245 | 1.523 | 1.190 | 1.212 | 0.937 | 0.630 | 0.506 | 0.232 | 0.146 |
|  | 1:200 | 2.207 | 1.202 | 1.539 | 0.958 | 1.207 | 1.054 | 1.213 | 0.528 | 0.705 | 0.488 | 0.243 | 0.211 |
|  | 1:400 | 2.070 | 1.162 | 1.216 | 0.745 | 0.911 | 0.710 | 0.922 | 0.728 | 1.051 | 0.363 | 0.239 | 0.195 |
|  | 1:800 | 1.742 | 1.178 | 1.017 | 0.649 | 0.824 | 0.552 | 0.814 | 0.493 | 1.049 | 0.278 | 0.253 | 0.191 |
|  | 1:1600 | 1.507 | 1.026 | 0.871 | 0.414 | 0.721 | 0.380 | 0.610 | 0.290 | 1.102 | 0.213 | 0.252 | 0.165 |
|  | 1:3200 | 1.194 | 0.768 | 0.781 | 0.302 | 0.579 | 0.265 | 0.477 | 0.228 | 1.299 | 0.255 | 0.183 | 0.114 |
|  | 1:6400 | 1.207 | 0.535 | 0.609 | 0.267 | 0.563 | 0.266 | 0.328 | 0.163 | 0.697 | 0.239 | 0.198 | 0.970 |
| pG-Tf2 | 1:50 | 2.452 | 1.194 | 2.225 | 1.265 | 1.722 | 1.156 | 1.213 | 0.831 | 1.203 | 0.786 | 1.112 | 0.621 |
|  | 1:100 | 2.638 | 1.149 | 2.154 | 1.276 | 1.692 | 1.150 | 1.150 | 0.746 | 1.141 | 0.665 | 0.876 | 0.529 |
|  | 1:200 | 2.226 | 1.273 | 1.868 | 0.997 | 1.510 | 0.856 | 1.151 | 0.649 | 1.037 | 0.499 | 0.626 | 0.428 |
|  | 1:400 | 1.828 | 0.847 | 1.745 | 0.815 | 1.320 | 0.743 | 0.832 | 0.508 | 0.873 | 0.483 | 0.431 | 0.316 |
|  | 1:800 | 1.395 | 0.763 | 1.349 | 0.615 | 1.245 | 0.565 | 0.650 | 0.488 | 0.632 | 0.464 | 0.324 | 0.270 |
|  | 1:1600 | 1.186 | 0.643 | 1.246 | 0.628 | 1.194 | 0.531 | 0.435 | 0.337 | 0.533 | 0.388 | 0.229 | 0.215 |
|  | 1:3200 | 0.833 | 0.468 | 0.943 | 0.462 | 0.861 | 0.436 | 0.345 | 0.265 | 0.301 | 0.277 | 0.215 | 0.142 |
|  | 1:6400 | 0.771 | 0.373 | 0.769 | 0.377 | 0.739 | 0.356 | 0.260 | 0.188 | 0.249 | 0.176 | 0.203 | 0.143 |
| pTf16 | 1:50 | 2.479 | 1.598 | 1.808 | 1.239 | 1.940 | 1.252 | 1.571 | 0.527 | 1.375 | 0.480 | 0.389 | 0.169 |
|  | 1:100 | 2.268 | 1.830 | 1.813 | 1.203 | 1.142 | 1.178 | 1.376 | 0.491 | 1.480 | 0.385 | 0.374 | 0.167 |
|  | 1:200 | 1.427 | 1.237 | 1.401 | 1.169 | 1.131 | 1.135 | 1.166 | 0.467 | 1.378 | 0.381 | 0.362 | 0.165 |
|  | 1:400 | 1.283 | 0.863 | 1.161 | 0.805 | 1.054 | 0.837 | 0.817 | 0.414 | 1.265 | 0.271 | 0.356 | 0.179 |
|  | 1:800 | 1.232 | 0.689 | 0.765 | 0.525 | 0.761 | 0.676 | 0.659 | 0.278 | 1.192 | 0.238 | 0.261 | 0.226 |
|  | 1:1600 | 0.813 | 0.556 | 0.756 | 0.448 | 0.582 | 0.382 | 0.571 | 0.346 | 1.224 | 0.218 | 0.273 | 0.209 |
|  | 1:3200 | 0.776 | 0.240 | 0.582 | 0.453 | 0.238 | 0.183 | 0.369 | 0.181 | 0.958 | 0.229 | 0.209 | 0.136 |
|  | 1:6400 | 0.521 | 0.294 | 0.395 | 0.38 | 0.279 | 0.230 | 0.186 | 0.120 | 0.835 | 0.223 | 0.195 | 0.121 |

C: Control well OD value, I: Inhibition well OD value


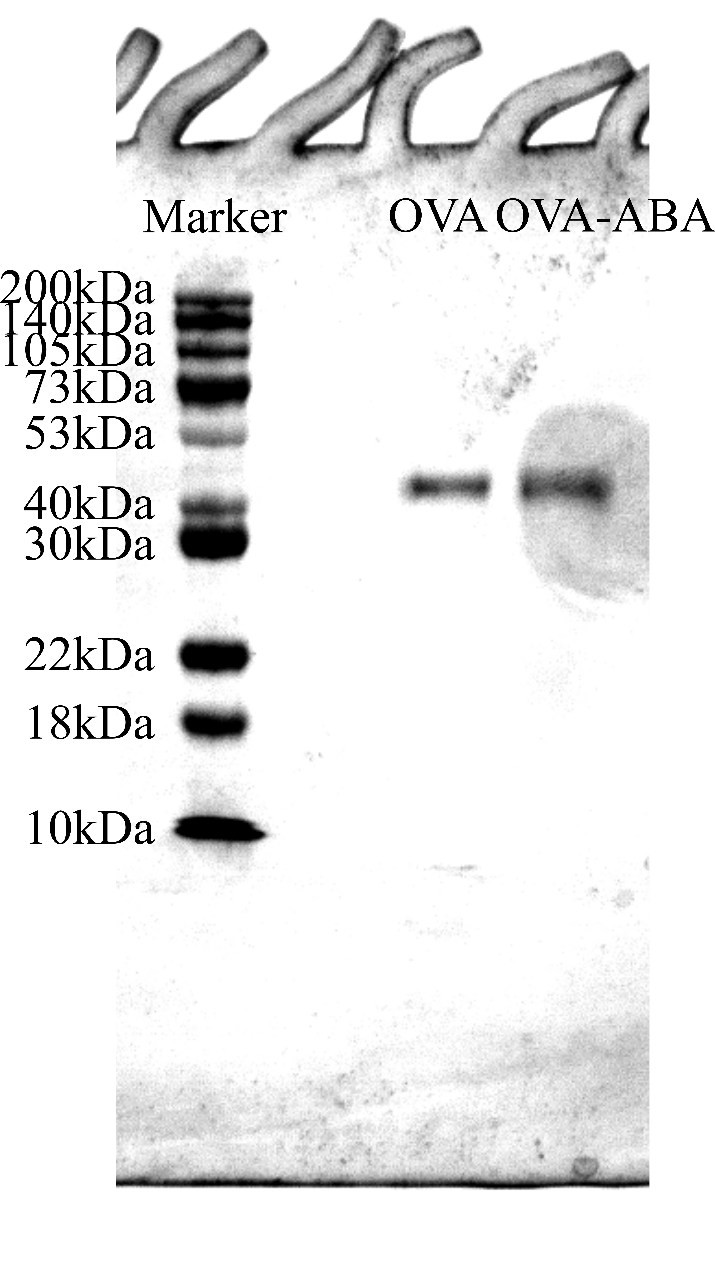


**Fig. S1** Purity assessment of OVA–ABA conjugates by SDS-PAGE.


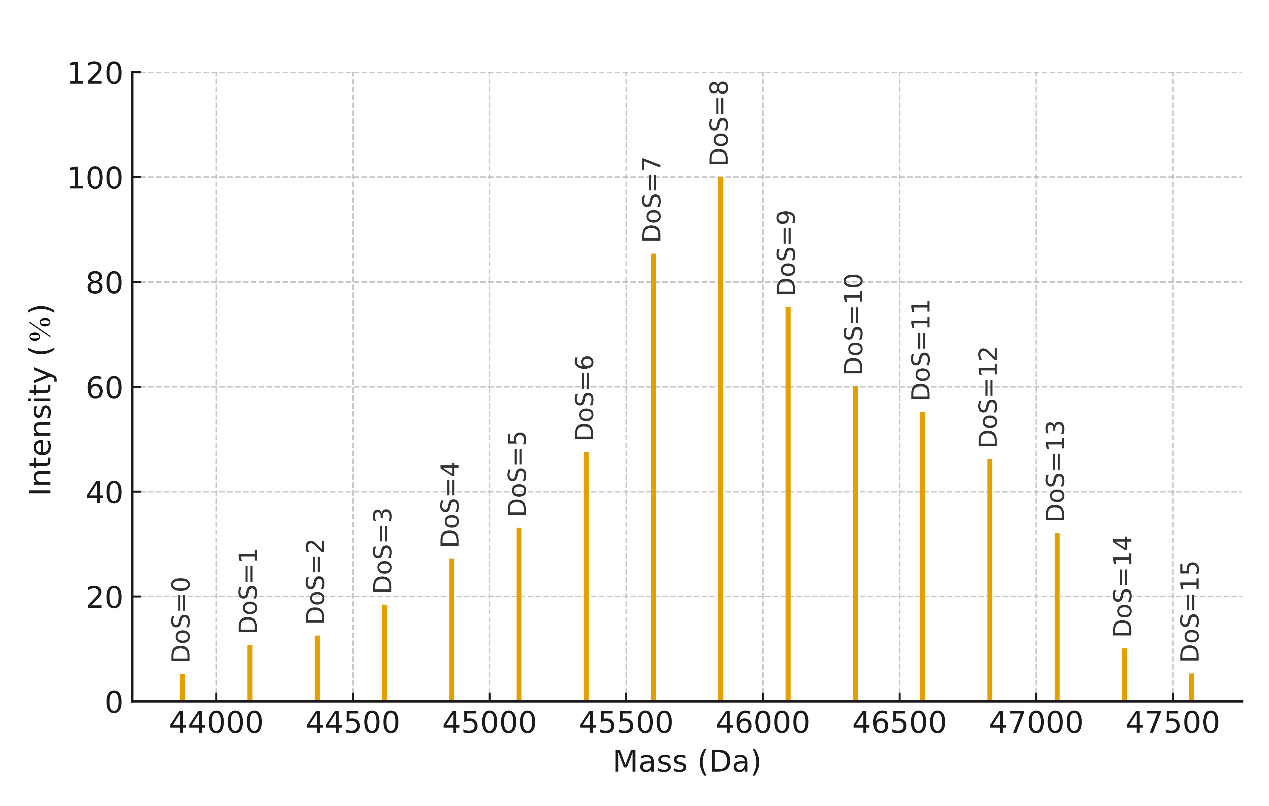


**Fig. S2** Deconvoluted qTOF-MS spectrum of OVA-ABA conjugates. Note: The labels indicate the Degree of Substitution (DoS) for each peak.


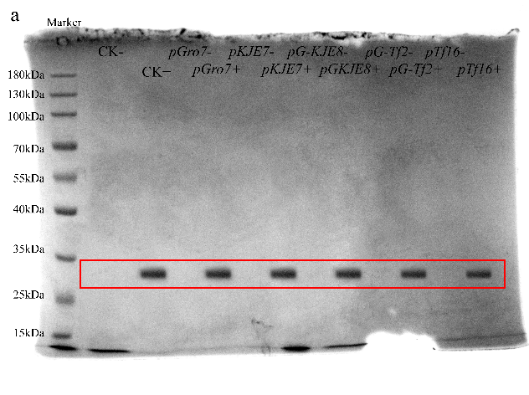

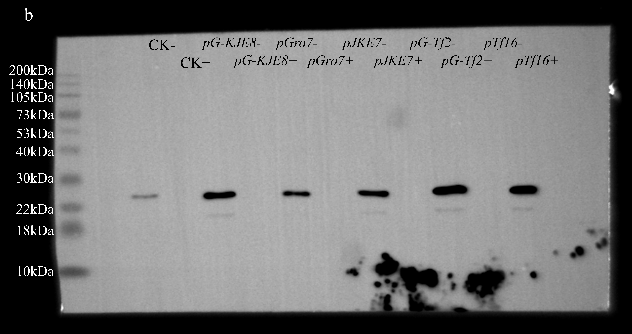


**Fig. S3** Fig. S3. SDS-PAGE (a) and Western blot (b) analysis of ABA-scFv expressed in IPTG-induced BL21(DE3) cells, showing distinct bands at the expected molecular weight (~26 kDa). Protein marker: SDS-PAGE, Servicebio, Cat# G2091-250UL; Western blot, Servicebio, Cat# G2058-250UL.


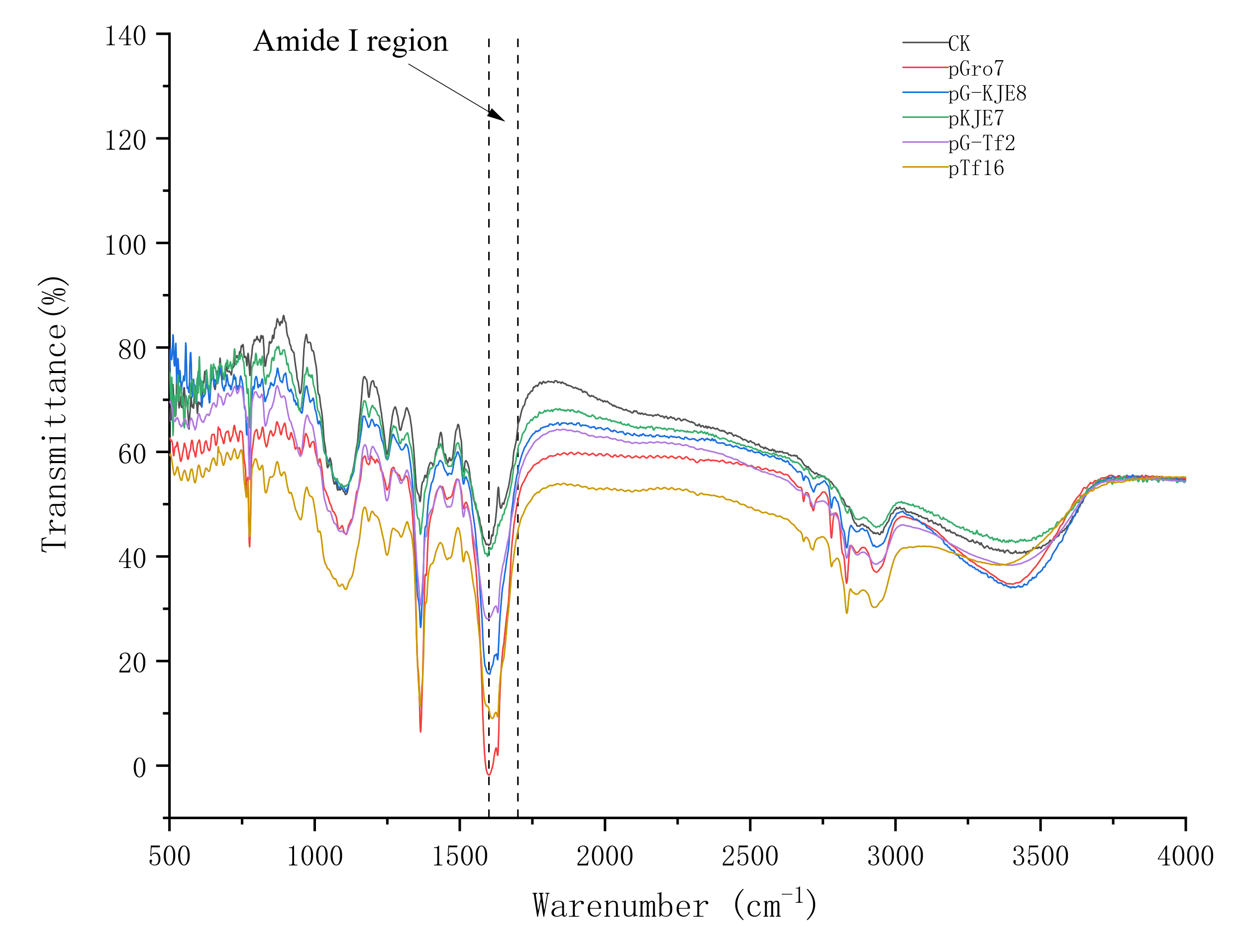


**Fig. S4** The FT-IR data of ABA Single-Chain Antibody.
